# Primary Liposarcoma of the Mediastinum

**DOI:** 10.1080/13577149877993

**Published:** 1998-12

**Authors:** Joel Greif, Silvia Marmor, Ofer Merimsky, Felix Kovner, Moshe Inbar

**Affiliations:** 1 Pulmonary Division Tel-Aviv Sourasky Medical Center and the Sackler Faculty of Medicine Tel-Aviv University Tel-Aviv Israel; 2 Pathology Department Tel-Aviv Sourasky Medical Center and the Sackler Faculty of Medicine Tel-Aviv University Tel-Aviv Israel; 3 Oncology Department Tel-Aviv Sourasky Medical Center and the Sackler Faculty of Medicine Tel-Aviv University 6 Weizman Street Tel-Aviv 64239 Israel

## Abstract

*Patient.* A 62-year-old man presented with effort dyspnea, non-productive cough and weakness of 4 month duration. He had no findings on physical examination.

*Discussion.* Chest X-ray revealed a large mass in the left anterior mediastinum. Computerized tomography of the chest showed a well-delineated homogeneous mediastinal mass with fat-equivalent density and a small pleural effusion. Fiberoptic bronchoscopy revealed narrowing of the left main bronchus, secondary to external compression. The bronchial mucosa was normal and brush cytology was negative. A CT-guided fine needle aspiration (FNA) of the mass yielded fragments of cells embedded in myxoid background material and closely packed atypical lipoblasts, compatible with liposarcoma. The patient underwent a left lateral thoracotomy and margibnal resection of the mass. The histopathological examination confirmed the diagnosis of mixed-type liposarcoma, consisted of myxoid and pleomorphic liposarcoma. Postoperative two-field radiation therapy was delivered to the mediastinum for a total midplane dose of 40 Gy. After a disease-free interval of 8 months the disease recurred in the mediastinum and pleura. Palliative chemotherapy achieved a short duration partial response but the patient succumbed to local recurrence 2 years after the diagnosis.


*Sarcoma* (1998) **2**, 205–207

CASE REPORT

### Primary liposarcoma of the mediastinum

JOEL GREIF,[1] SILVIA MARMOR,[2] OFER MERIMSKY,[3] FELIX KOVNER[3] & MOSHE INBAR[3]

[1]*Pulmonary Division,* [2]*Pathology Department and* [3]*Oncology Department, Tel-Aviv Sourasky Medical Center and the Sackler Faculty of Medicine, Tel-Aviv University, Tel-Aviv, Israel*

**Abstract**

*Patient.* A 62-year-old man presented with effort dyspnea, non-productive cough and weakness of 4 month duration. He had no findings on physical examination.

*Discussion.* Chest X-ray revealed a large mass in the left anterior mediastinum. Computerized tomography of the chest showed a well-delineated homogeneous mediastinal mass with fat-equivalent density and a small pleural effusion. Fiberoptic bronchoscopy revealed narrowing of the left main bronchus, secondary to external compression. The bronchial mucosa was normal and brush cytology was negative. A CT-guided fine needle aspiration (FNA) of the mass yielded fragments of cells embedded in myxoid background material and closely packed atypical lipoblasts, compatible with liposarcoma. The patient underwent a left lateral thoracotomy and margibnal resection of the mass. The histopathological examination confirmed the diagnosis of mixed-type liposarcoma, consisted of myxoid and pleomorphic liposarcoma. Postoperative two-field radiation therapy was delivered to the mediastinum for a total midplane dose of 40 Gy. After a disease-free interval of 8 months the disease recurred in the mediastinum and pleura. Palliative chemotherapy achieved a short duration partial response but the patient succumbed to local recurrence 2 years after the diagnosis.

**Key words:** *primary liposarcoma, mediastinum, neoplasm.*

### Introduction

Liposarcomas are relatively uncommon neoplasms, accounting for approximately 15–20% of all sarcomas.[1] They usually arise in the lower extremities or retroperitoneum but have been reported in other locations such as the abdomen, vulva and buttocks. Primary lipoblastic tumors originating in the mediastinum are extremely rare.

The present report concerns a patient with such a primary mediastinal liposarcoma first diagnosed by fine needle aspiration biopsy. The clinical course, pathological findings and therapeutic approaches are presented and the current literature is reviewed.

### Case Report

A 62 year-old man presented with dyspnea on effort, non-productive cough and weakness of 4 months' duration. The physical examination was unremarkable and blood count and biochemistry values were normal. A chest X-ray revealed a large anterior mass in the left anterior mediastinum (Fig. 1(a)). The computerized tomography (CT) of the chest (Fig. 1(b)) showed a well-delineated mass in the anterior mediastinum in the vicinity of the heart and an associated small pleural effusion on the same side, which was lapped and found to be without malignant cells. The mass was homogeneous end its density was compatible with fat. Abdominal CT was normal. Fiberoptic bronchoscopy revealed narrowing of the left main bronchus, secondary to external compression. The bronchial mucosa was normal and brush cytology was negative. A CT-guided fine needle aspiration biopsy (FNA) of the mass yielded fragments of cells embedded in myxoid background material as well as closely packed atypical lipoblasts, compatible with liposarcoma. The patient underwent a left lateral thoracotomy in order to better expose the pericardium and the tumoral mass, and to have a better control on possible pulmonary invasion. An anterior mediastinal mass of soft consistency invading the adjacent pericardium, but not the chest wall, was marginally excised together with a portion of the involved pericardium. Five hundred cc of bloody pleural fluid were also drained and the cytological findings were negative. The lung parenchyma was not involved. The left lung fully expanded after surgery and the postoperative period

Correspondence to: O. Merimsky, Department of Oncology, Tel Aviv Sourasky Medical Center, 6 Weizman Street, Tel-Aviv 64239, Israel. Tel.: Int-972-3-697-4830; Fax: Int-972-3-697-4828.

1357-714X/98/030205-03 $9.00 © 1998 Carfax Publishing Ltd was uneventful. Pathological examination of the operative specimen showed a well-circumscribed mass, 10 cm in diameter and gelatinous in appearance. The histopathological examination showed areas of proliferating lipoblasts in varying stages of differentiation, with a delicate plexiform capillary pattern and a myxoid matrix containing hyaluronidase-sensitive mucopolyssocharides. Other areas showed a disorderly growth pattern and an extreme degree of cellular pleomorphism including bizarre cells. These features are compatible with a mixed-type liposarcoma (Fig 1(c)). Following surgery, due to pericardial involvement and tumor marginal excision, the patient received external radiation to the anterior mediastinum for a total dose of 4000 cGy (AP-PA filed, 180 cGy $\times$ 5/week). Follow-up included medical history and chest plain film every 3 months, and chest CT every 6 months. He remained asymptomatic for a disease-free period of 8 months, when he complained of dyspnea and chest pain. A repeated CT demonstrated a recurrent anterior mediastinal mass and irregular thickening of the pleura. Fine needle aspiration biopsy of the recurrent mediastinal mass yielded liposarcoma cells. A trial of chemotherapy, including Cyclophosphamide, Oncovin, Adriamycin and Ifosfamide, was only partially successful. The patient succumbed to his disease 2 years after the appearance of the symptoms and discovery of the primary tumor.

### Discussion

Liposarcoma is a relatively common tumor of the lower legs, posterior peritoneum and gluteal region and accounts for 15–20% of all soft tissue malignant tumors. Mediastinal location is very unusual: Indeed, recent reviews of the literature described only about 90 cases of primary mediastinal liposarcomas.[2–6] The age range of the patients was reported as being between 20 and 70 years with a peak in the 5th decade, and there were no differences between the sexes. One of the more typical characteristics of mediastinal liposarcoma is its ability to attain a very large size yet remain undetected. Patients may be asymptomatic and diagnosed incidentally on a routine chest roentgenogram. In CT examination liposarcomas usually have a homogeneous appearance with attenuation levels similar to or much higher than normal fat.[7] Clinical symptoms are due mostly to compression of mediastinal structures, causing respiratory distress, chest pain and cough, such as in our patient.

Among the patients reviewed by Schweitzer and Aguam,[5] 63% presented with respiratory symptoms, 50% with chest pain and 15% with superior vena cava obstruction.[8]Macroscopically, liposarcomas are circumscribed, sometimes encapsulated lobulated masses which infiltrate adjacent soft tissues and viscera. Enzinger and Weiss[1] classified liposarcomas into five histopathological types:

1. well-differentiated liposarcoma;
2. myxoid liposarcoma;
3. round cell or adenoid type;
4. pleomorphic liposarcoma; and
5. mixed group comprising 2 or more elements (as in our case).

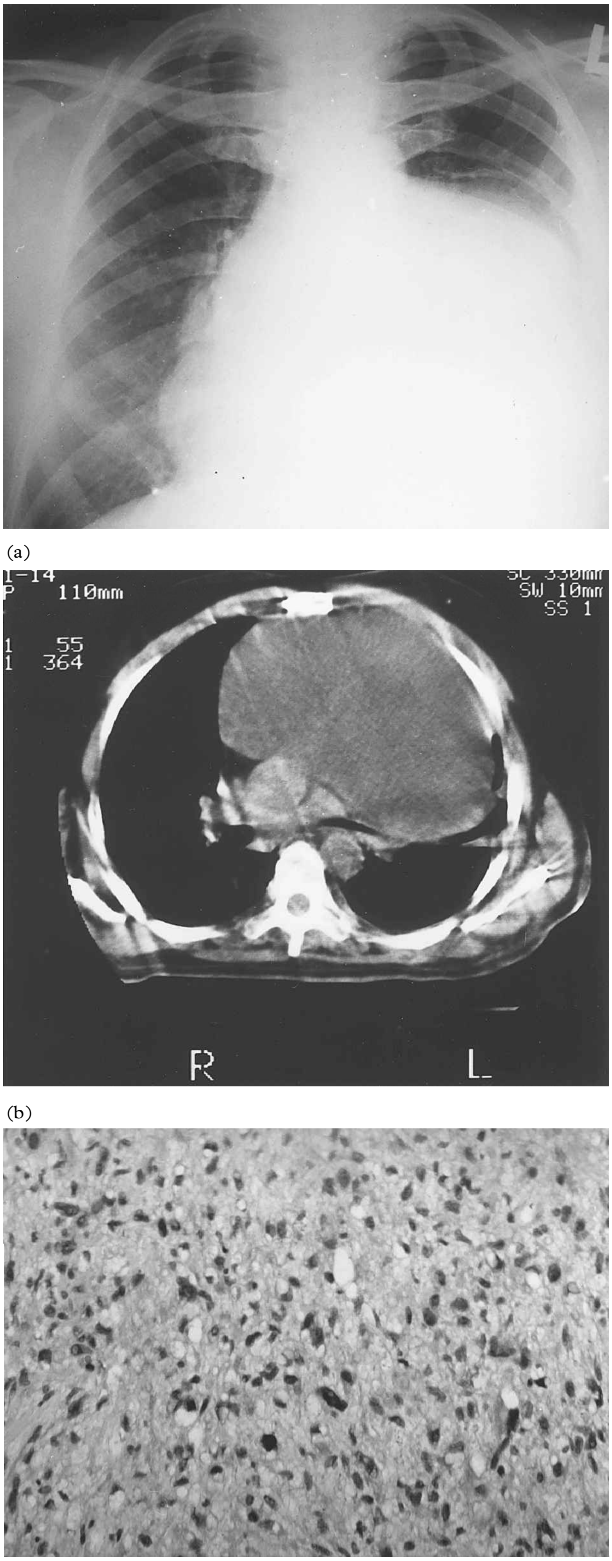

**Fig. 1.** *Chest plain film (a) and computerized tomography (b) of the chest showing a large anterior mediastinal mass, found to be (c) mixed (myxoid and pleomorphic) liposarcoma* (H&E $\times$ 1000).

The clinical course is characterized by frequent recurrence of the tumor and death due to local disease, with a mean survival of 2.5 years.[4] Fine needle aspiration will reveal a characteristic picture and help to establish the diagnosis[9,10]—as in our case.

Because of the low incidence of primary mediastinal sarcomas, treatment strategies are extrapolated from similar tumors in other places. The first choice of therapy for mediastinal liposarcoma is surgical excision of the tumor; but, because of invasion to surrounding tissues total excision is frequently impossible.

Patient survival following surgery is closely related to histological type. In cases of well-differentiated tumors, the surgery can be curative in nearly all patients. With poorly differentiated histological types and invasion of adjacent organs, surgery is mainly palliative, with the removal of the bulk of the tumor allowing only symptomatic relief. Radiotherapy is the treatment of choice for unresectable tumors or in the event of local recurrence.[8]

Reported cases of good therapeutic responses to high-dose radiation treatment of liposarcomas in other locations[11,12] stand in marked contrast to the poor results reported in mediastinal tumors. It should be mentioned that such large doses (up to 9000 rad) as used in peripheral tumors would carry the risk of mediastinal and paricardial fibrosis.

The role of chemotherapy has yet to be determined. Based on the experience with limb lesions, the addition of chemotherapy could be considered in cases of high-grade malignant histologies or incomplete resected lesions. Whether such therapy will have an impact on survival is, as yet, unknown at this time. Some reports suggest that chemotherapy is effective against this disorder,[12,13] while others found it not to be useful.[14]

In conclusion, primary mediastinal liposarcomas are very rare. A presumptive diagnosis of this tumor can be obtained by chest CT and confirmed by percutaneous needle biopsy. Principles of treatment include surgical resection and adjuvant radiochemotherapy. Despite multimodality therapy, the prognosis continues to be poor.

### References

1 Enzinger FM, Weiss SW. *Liposarcoma: in soft tissue tumors*. St Louis: CV Mosby, 1983:242–80.

2 Dogan R, Ayrancioglu K, Aksu O. Primary mediastinal liposarcoma. A report of a case and review of the literature. *Eur J Cardiothorac Surg* 1989; 3:357–70.

3 Grewal RG, Prager K, Austin JM, Rotterdam H. Long-term survival in non-encapsulated primary liposarcoma of the mediastinum. *Thorax* 1993; 48:1276–7.

4 Klimstra DS, Moran CA, Perino G, Koss MN, Rosal J. Liposarcoma of the anterior mediastinum and thymus—a clinicopathological study of 28 cases. *Am J Surg Pathol* 1995; 19:782–91.

5 Schweitzer DL, Aguam AS. Primary liposarcoma of the mediastinum. Report of a case and review of the literature. *J Thorac Cardiovasc Surg* 1977; 74:83–97.

6 Shibata K, Koga Y, Onitsuka T, Wake N, Ishil K, Sekiya P. Sumyoshi A. Primary liposarcoma of the mediastinum—a case report and review of the literature. *Jpn J Surg* 1986; 16:277–83.

7 Santamaria G, Serres X. Prune X. Primary mediastinal liposarcoma with moderately high CT attenuation. *AJR* 1996; 1064–5.

8 Reltan JB, Kaalhus O. Radiotherapy of liposarcomas. *Br J Radiol* 1980; 53:969–75.

9 Attal H, Jensen JoA, Reyes CV. Myxoid liposarcoma of the anterior mediastinum. Diagnosis by fine needle aspiration biopsy. *Acta Cytol* 1995; 39:511–3.

10 Shattuck MC, Victor TA. Cytologic features of well-differentiated sclerosing liposarcoma in aspirated samples. *Acta Cytol* 1988; 32:896–901.

11 Friedman M, Egan JW. Effect of irradiation on liposarcoma. *Acta Radiol* 1960; 64:225–31.

12 James DH. Effective chemotherapy for abdominal liposarcoma. *Pediatrics* 1986; 63:311–5.

13 Antman KA, Blum RH, Wilson RE. Survival of patients with localized high-grade soft tissue sarcoma with multimodality therapy. *Cancer* 1983; 51:396–40l.

14 Standerfer RJ, Armistead SH, Paneth M. Liposarcoma of the mediastinum report of two cases and review of the literature. *Thorax* 1981; 36:693–4.
